# Is endoscopic surveillance necessary for patients who undergo total gastrectomy for gastric cancer?

**DOI:** 10.1371/journal.pone.0196170

**Published:** 2018-06-01

**Authors:** Sung Jae Park, Young Soo Park, In Sub Jung, Hyuk Yoon, Cheol Min Shin, Sang-Hoon Ahn, Do Joong Park, Hyung Ho Kim, Nayoung Kim, Dong Ho Lee

**Affiliations:** 1 Department of Internal medicine, Seoul National University Bundang Hospital, Seongnam-si, Republic of Korea; 2 Department of Internal medicine, Seoul Medical Center, Seoul, Republic of Korea; 3 Department of Surgery, Seoul National University Bundang Hospital, Seongnam-si, Republic of Korea; University Hospital Llandough, UNITED KINGDOM

## Abstract

There have been only a few reports investigating the clinical efficacy of follow-up endoscopy for detection of recurrent gastric cancer after total gastrectomy (TG). We reviewed the records of 747 patients undergoing TG from 2003 to 2012 and enrolled 267 patients (70 with early gastric cancer (EGC) and 197 with advanced gastric cancer (AGC)), who received one or more follow-up endoscopy and contrast abdominal computed tomography (CT) scan. We found no tumor recurrence in the 70 EGC patients during the mean follow-up periods of 42.1 ± 18 and 43.2 ± 19 months by endoscopy and contrast abdominal CT scan. In 197 AGC patients, 59 patients (29.8%) had confirmed tumor recurrence during mean follow-up periods of 40.5 ± 21 and 45.3 ± 22 months. The most common pattern of tumor recurrence was distant metastasis (n = 35) followed by peritoneal metastasis (n = 11). Among the other 13 cases with loco-regional recurrence, seven cases were regional lymph node metastases, four were anastomosis site recurrences, and two were duodenal stump and jejunal loop site recurrences. Three of the four cases of anastomosis site recurrence were found by both endoscopy and contrast abdominal CT scan; one case was missed by contrast abdominal CT scan. However, the two cases with duodenal stump and jejunal loop recurrences were detected by contrast abdominal CT scan only. An annual follow-up endoscopy for gastric cancer after TG might have a limited role in the detection of tumor recurrence, especially in patients with EGC. Contrast abdominal CT scan may be sufficient as a follow-up method for recurrent gastric cancer after TG.

## Introduction

The overall clinical outcome of gastric cancer has improved because of growing recognition of potential carcinogens, the development of health screening programs for early detection of gastric cancer, and improvements in surgical and multimodality treatments. Nevertheless, the overall survival of patients with advanced gastric cancer (AGC) receiving radical gastrectomy is low, and most recurrent cases arise within the first 5 years after radical gastrectomy [1]. Although the overall rate of tumor recurrence is reported to be low, death due to blood-borne metastasis to another organ and lymph node metastasis are sometimes observed in patients with early gastric cancer (EGC) receiving curative gastrectomy [2–4]. Therefore, many clinicians perform intensive post-operative follow-up or surveillance of patients with gastric cancer who have undergone curative surgery [5, 6], even though there is no clear clinical evidence that it improves outcomes or survival. There is also no consensus on the methods of surveillance for these patients.

Most currently published oncology guidelines aim to detect early recurrent disease and improve patients’ quality of life. However, they lack details on the methods, duration, and intensity of surveillance. For example, The National Cancer Comprehensive Network (NCCN) guidelines suggest that all patients with gastric cancer should undergo follow-up or surveillance after surgery, including a complete history taking and physical examination every 3–6 months for the first 1–2 years, every 6–12 months for the next 3–5 years, and then annually. In contrast, other examinations, such as complete blood count, chemistry profile, radiologic imaging, and endoscopy are recommended when clinically indicated by symptoms [7]. In contrast, The European Society of Medical Oncology guidelines and the British and Scottish guidelines both state that no clinical evidence exists to support the idea that regular follow-up or surveillance programs improve overall survival outcomes, even though regular follow-up may allow treatment of symptoms, provide psychological support, and assist in early detection of recurrent tumors. Therefore, they recommend symptom-driven outpatient visits and directed investigations only in patients with symptoms and who are candidates for further treatment [8,9].

However, some authors suggest that follow-up endoscopic surveillance for early detection of gastric remnant cancer could be helpful for patients receiving curative gastric cancer surgery [10,11]. But, the role and clinical efficacy of follow-up endoscopy after total gastrectomy (TG) has not been established, because there is no remnant gastric mucosa. Nevertheless, many institutions perform annual follow-up endoscopic surveillance after TG without any definite clinical evidence to support the practice.

In Korea, follow-up endoscopic and contrast abdominal computed tomographic (CT) surveillance have been routinely performed together. In this study, we have evaluated the clinical efficacy of routine follow-up endoscopic surveillance for gastric cancer after TG.

## Materials and methods

### Patients

We retrospectively reviewed the medical records of 747 patients diagnosed with gastric cancer (EGC and AGC) who had undergone TG with R0 resection from March 2003 to December 2012 at Seoul National University Bundang Hospital. From the total 747 patients, we enrolled 267 patients who received one or more periodic follow-up endoscopies and contrast abdominal CT scans. Patients with TG for palliation, recurrence after subtotal gastrectomy (STG), endoscopic resection, or double primary cancers were excluded.

### The type of tumor recurrence based on endoscopic and CT finding

We investigated patients’ and tumor basal characteristics, treatment modality, follow-up period and status after TG, follow-up endoscopic and contrast abdominal CT scan findings, and tumor recurrence pattern. The type of tumor recurrence was divided into three groups [12]: (1) distant tumor recurrence, (2) peritoneal tumor recurrence, and (3) loco-regional tumor recurrence. Loco-regional tumor recurrence included both endoscopic-accessible tumor recurrence (at the anastomosis site or within intestinal loop) and endoscopic-inaccessible tumor recurrence (enlarged regional lymph nodes or a mass near the resected site). The gastric cancers were staged according to the American Joint Committee on Cancer staging (AJCC 7^th^ edition) tumor node metastasis (TMN) classification.

### Statistical analysis

Student’s *t-*test and the chi-squared test were used for the statistical assessment of continuous and categorical variables. A *p*-value less than 0.05 was considered as statistically significant. IBM SPSS Statistics ver. 22 was used to perform the analyses.

### Ethics statement

The study protocol was approved by the institutional review board at Seoul National University Bundang Hospital (IRB No: B-1511-322-108). This study is a retrospective study that states that the IRB committee has waived its consent form because it could not receive the consent form in a realistic way and does not have a significant impact on the subject.

## Results

### Baseline characteristics of patients and tumors

A total of 267 patients (70 EGC patients and 197 AGC patients) were enrolled. Baseline characteristics of the patients and their tumors are listed in Tables 1 and 2. The mean age of the patients was 58.8 (27–85) years, and the male to female ratio was 2.4:1. The mean age and male to female ratio in the EGC and AGC groups was 59.2 (27–85) years and 2.2:1, and 58.7 (29–83) years and 2.5:1, respectively. No statistically significant difference was found between the EGC and the AGC group for the mean age, sex ratio, smoking history, final education level, family history of malignancy, or tumor site. However, statistically significant difference was found between the two groups for body mass index (BMI) (*p* = 0.012). Also, the mean tumor size was larger in the AGC group than in the EGC group.

**Table 1:** Baseline characteristics of the patients.

	EGC (n = 70)	AGC (n = 197)	*p*-value
**Mean age (years old)**	59.2 (27–85)	58.7 (29–83)	0.744
**Sex (male/female)**	48/22 (2.2:1)	140/57 (2.5:1)	0.892
**BMI (Weight(Kg)/Height(m**^**2**^**))**	21.8 (16.2–35.4)	20.7 (12.1–36.8)	0.012
**Smoking History**			0.931
Never smoker	48 (68.6%)	133 (67.5%)	
Past smoker	7 (10.0%)	12 (6.1%)	
Current smoker	15 (21.4%)	52 (26.4%)	
**Education level**			0.238
No education	9 (12.9%)	6 (3.0%)	
Elementary school	1 (1.4%)	42 (21.3%)	
Middle school	10 (14.3%)	26 (13.2%)	
High school	19 (27.1%)	62 (31.3%)	
College or University	31 (44.3%)	61 (31.0%)	
**Family history of malignancy**			0.59
Gastrointestinal	15 (21.4%)	34 (17.3%)	
Hepatopancreaticobiliary	2 (2.9%)	10 (5.1%)	
Respiratory	0 (0%)	5 (2.5%)	
Urogenital	2 (2.9%)	4 (2.0%)	
Hematology	3 (4.3%)	0 (0%)	
Breast	2 (2.9%)	5 (2.5%)	
Unspecified	0 (0%)	2 (1.0%)	
None	46 (65.7%)	137 (69.5%)	

EGC, early gastric cancer; AGC, advanced gastric cancer; BMI, body mass index.

**Table 2:** Baseline characteristics of the tumors.

	EGC (n = 70)	AGC (n = 197)	*p*-value
Gross type	Total 76 lesion	Total 197 lesion	NA
I / Borrmann type I	3 (3.9%)	12 (6.1%)	
IIA / Borrmann type II	5 (6.6%)	30 (15.2%)	
IIB / Borrmann type III	7 (9.2%)	116 (58.9%)	
IIC / Borrmann type IV	43 (56.6%)	37 (18.8%)	
III	0 (0%)		
Mixed / Unclassified	18 (23.6%)	2 (1.0%)	
**Size of tumor (cm)**	3.1 x 2.3	6.7 x 5.2	0.001
**Site of tumor**			0.782
Upper third	59 (77.6%)	175 (88.8%)	
Middle third	16 (21.1%)	12 (6.1%)	
Lower third	1 (1.3%)	0 (0%)	
Entire	0 (0%)	10 (5.1%)	
**Number of tumor**			
Single	64 (91.4%)	197 (100%)	
Multiple	6 (8.6%)	0 (0%)	
**Stage**			NA
IA	66 (94.3%)	0 (0%)	
IB	1 (1.4%)	36 (18.3%)	
IIA	2 (2.9%)	55 (27.9%)	
IIB	1 (1.4%)	34 (17.3%)	
IIIA	0	27 (13.7%)	
IIIB	0	23 (11.7%)	
IIIC	0	21 (10.7%)	
IV	0	1 (0.5%)	
**Histology**			
Well differentiated	11 (14.5%)	1 (0.5%)	
Moderate differentiated	27 (35.5%)	55 (27.9%)	
Poorly differentiated	24 (31.6%)	92 (46.7%)	
Signet ring cell	12 (15.8%)	41 (20.8%)	
Mixed	2 (2.6%)	1 (0.5%)	
Unclassified	0	7 (3.6%)	

EGC, early gastric cancer; AGC, advanced gastric cancer.

### Treatment modalities of both EGC and AGC groups

The 70 EGC patients and 197 AGC patients were treated with TG with R0 resection, and most tumors were located in the upper third of the stomach (Table 2). In the EGC group, only one patient had a tumor located in the lower third of stomach. The patient had requested TG because of a family history of gastric cancer and patient’s fear of tumor recurrence on the remnant gastric tissue. Three patients in the EGC group and 130 patients in the AGC group had received chemotherapy (CTx) after TG. Seven patients in the EGC group and one in the AGC group had received both radiotherapy (RTx) and concurrent chemotherapy (CCRT) before or after TG (Table 3).

**Table 3:** Adjuvant therapy of both EGC and AGC.

	EGC (n = 70)	AGC (n = 197)
**Chemotherapy**	3 (4.3%)	130 (66.0%)
**Radiotherapy**	0	7 (3.6%)
**CCRT**	0	1 (0.5%)

EGC, early gastric cancer; AGC, advanced gastric cancer; CCRT: concurrent chemoradiotherapy.

### Type of tumor recurrence

The mean follow-up period, the follow-up status of patients, and the patterns of tumor recurrence are shown in Tables 4 and 5. In 70 EGC patients, we found no tumor recurrence during the mean follow-up periods of 42.1 ± 18 and 43.2 ± 19 months by endoscopy and contrast abdominal CT scan, respectively. The mean number of follow-up endoscopy and contrast abdominal CT scan performed was 4 and 4.1 per person, respectively. In 197 AGC patients, 59 patients (29.9%) were confirmed as having a tumor recurrence during the mean follow-up periods of 40.5 ± 21 and 45.3 ± 22 months by endoscopy and contrast abdominal CT scan, respectively. The mean number of follow-up endoscopy and contrast abdominal CT scan performed was 3.9 and 7.9 per person, respectively.

**Table 4:** The mean follow-up period and the follow-up status of both patients.

	EGC (n = 70)	AGC (n = 197)	*p*-value
**Endoscopy**	42.1 ± 18 months	40.5 ± 21 months	0.597
**Abdominal CT scan**	43.2 ± 19 months	45.3 ± 22 months	0.48
**Follow-up finished (Cured status)**	29 cases (41.4%)	41 cases (20.8%)	
**OPD follow-up**	29 cases (41.4%)	76 cases (38.6%)	
**Transfer-out to other hospitals**	4 cases (5.7%)	16 cases (8.1%)	
**Follow-up loss**	7 cases (10.0%)	36 cases (18.3%)	
**Death**	1 cases (1.4%)	28 cases (14.2%)	

EGC, early gastric cancer; AGC, advanced gastric cancer; Abdominal CT, Abdominal computed tomography.

**Table 5:** The pattern of tumor recurrence.

	EGC (n = 0)	AGC (n = 59)
**Loco-regional**	0	13 (22.0%)
Regional Lymph node	0	7 (53.8%)
Anastomosis	0	4 (30.8%)
Intestinal loop	0	2 (15.4%)
**Peritoneal**	0	11 (18.6%)
**Distant**	0	35 (59.3%)

EGC, early gastric cancer; AGC, advanced gastric cancer.

The most common pattern of tumor recurrence was distant metastasis (n = 35), followed by peritoneal metastasis (n = 11). Among the remaining 13 cases with loco-regional recurrence, seven cases were regional lymph node metastasis, four were anastomosis site recurrence, and two were duodenal stump and jejunal loop site recurrences. Three of the four cases of anastomosis site recurrence were found by both follow-up endoscopy and contrast abdominal CT scan, but the other case was missed on follow-up contrast abdominal CT scan. In this case, unlike the other cases, endoscopy and contrast abdominal CT scan were performed on different days (the CT scan was performed about 10 days prior) and the tumor recurred as a slightly elevated mucosa and hyperemic erosion, which was sampled by endoscopic biopsy. However, the two cases with duodenal stump and jejunal loop recurrence were detected by contrast abdominal CT scan only.

### Endoscopic and contrast abdominal CT findings of peri-anastomotic tumor recurrence in patients diagnosed with AGC

**Fig 1:**
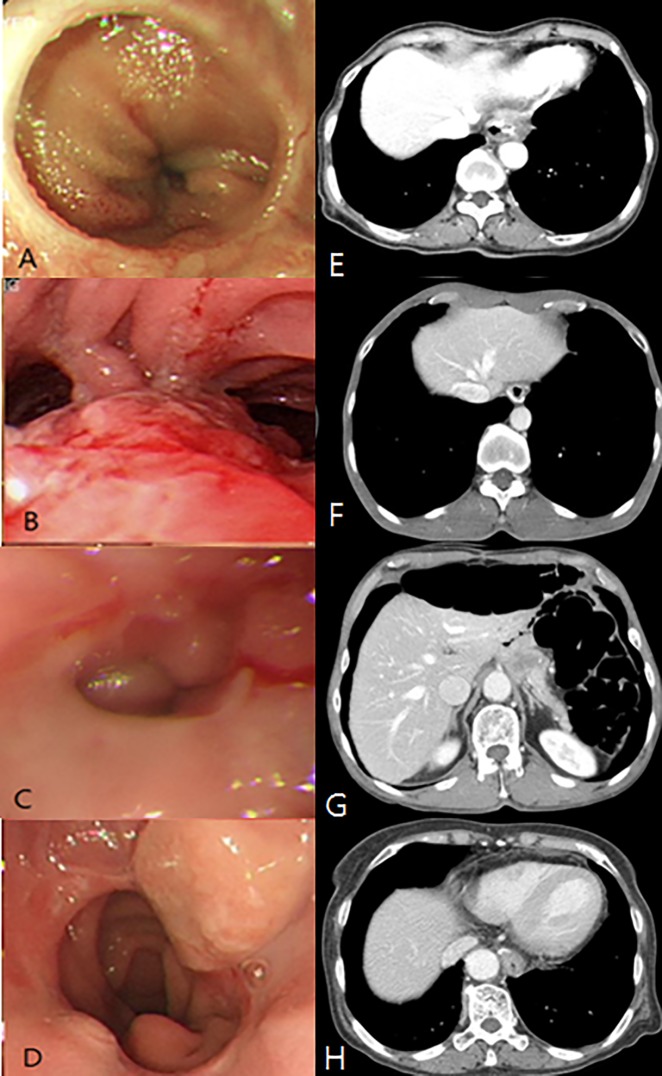
Endoscopic and contrast abdominal CT findings of peri-anastomotic tumor recurrence in patients diagnosed with AGC (each horizontal row of photos are from the same patient). A. Obstruction below the anastomosis site due to external compression B. Hyperemic and slightly elevated erosion, below the anastomosis site (missed by follow-up contrast abdominal CT scan) C. Stricture of the anastomosis site D. Subepithelial-like mass lesion, above the anastomosis site E. Prominent soft tissue density around the peri-anastomosis site F. No definite abnormal findings around the anastomosis site (missed by follow-up contrast abdominal CT scan) G. Low attenuation wall thickening at the anastomosis site H. Mild wall thickening just above the anastomosis site.

The endoscopic and contrast abdominal CT findings of peri-anastomotic tumor recurrence in patients diagnosed with AGC are shown in Fig 1. The most common endoscopic finding indicating a recurrence was a mass lesion (three patients) or stenosis (one patient). Four patients in the AGC group had tumor recurrence at the peri-anastomosis site, and of these, three cases were detected by both follow-up endoscopy and contrast abdominal CT scan. The other case was missed on a follow-up contrast abdominal CT scan. The only difference for this patient was the timing of the two follow-up methods. For the first three cases, they were performed on the same day, but the other case had a contrast abdominal CT scan performed about 10 days prior to endoscopy.

### The characteristics and clinical outcomes of AGC patients with peri-anastomotic tumor recurrence

Among the four AGC patients with peri-anastomosis site recurrence (a tumor recurrence after an average of 14 months after TG), two patients had been getting a poor performance status during OPD follow-up after TG because of old age and comorbidities, since then, further follow-up examinations and other treatments such as palliative surgery, CTx, or RTx were not performed, and they died. Another patient was treated with palliative CTx, RTx, and endoscopic stent insertion due to upper gastrointestinal obstruction, but she died of hemorrhagic shock due to cancer bleeding. The final patient underwent esophageal segmental resection and reconstruction of the Roux en Y esophagojejunostomy, and was subsequently transferred to another hospital due to a residence problem. He was lost to follow-up. Detection of tumor recurrence in patient 2 was missed on abdominal CT. A summary of the four patients is listed in Table 6.

**Table 6:** AGC patients with anastomosis site recurrence.

	Patient			
	Patient 1	Patient 2	Patient 3	Patient 4
**Age/Sex**	81/F	52/M	71/M	69/F
**Tumor site**	Upper third	Upper third	Upper third	Upper third
**Tumor size**	6.5 x 4.5 cm	8.3 x 7.9 cm	5.9 x 4.8 cm	7 x 5 cm
**Gross type**	Borrmann III	Borrmann III	Borrmann III	Borrmann III
**Histology**	SRC	PD	MD	SRC
**Stage**	IIB	IIB	IIIC	IIIC
**Adjuvant treatment**	None	Re-op and CTx	None	CTx and RTx
**Final clinical outcome**	Death	follow-up loss	Death	Death

SRC, signet ring cell; MD, moderate differentiated; PD, poorly differentiated; CTx, chemotherapy; RTx, radiotherapy. Detection of recurrence of tumor in patient 2 was missed on abdominal CT

## Discussion

Today, minimally invasive treatments such as endoscopic mucosal resection (EMR) or endoscopic submucosal dissection (ESD) are popular among patients diagnosed with gastric cancer. Nevertheless, many gastric cancer patients are treated with surgery, especially TG because of the site, the number, and the histological type of the gastric cancer [13–17]. However, an appropriate follow-up or surveillance strategy after EMR, ESD, and gastric surgery based on recurrence incidence, time intervals and patterns has not been established. Several guidelines suggest using surveillance, but they lack details on the methods, duration, and intensity of surveillance.

Many studies have mentioned the necessity of a follow-up endoscopy for detection of remnant gastric cancer after STG and of a follow-up contrast abdominal CT scan to search for metachronous cancers and lymph node or distant organ metastasis. Takeda et al. in their retrospective study reported the clinical efficacy of periodic endoscopic examinations for patients with partial gastrectomy for EGC, suggesting that to improve the result of surgical management of recurrent gastric cancer of the remnant-stump, early diagnosis is the most important factor. Therefore, periodic endoscopic surveillance after partial gastrectomy, especially around the anastomosis site, is necessary [18].

However, Yasuhiro et al. suggested that a close follow-up program, including annual endoscopy to search for recurrent gastric cancer in the gastric remnant mucosa, beginning 1–1.5 years after gastric surgery, and abdominal ultrasound (US) or CT scan performed every 6 months for at least 5 years may offer no survival benefit until the development of more effective treatment modalities, despite the early detection of asymptomatic gastric cancer recurrence in their retrospective study [19]. Also, Roberta et al. analyzed five studies from January 1^st^ 1998 to December 1^st^ 2009 in their systematic review of patient surveillance after curative gastrectomy for gastric cancer. They concluded that a more intense surveillance such as endoscopy and contrast abdominal CT scan failed to produce an improvement in the overall survival rate of patients [20].

As described above, the clinical efficacy of the follow-up programs after a gastric cancer surgery, such as endoscopy and contrast abdominal CT scan, is controversial. There are very few studies exploring the necessity or efficacy of follow-up endoscopy or contrast abdominal CT scan after TG, especially after TG with R0 resection. Lee et al. stated in their retrospective study that follow-up endoscopy after TG for detection of recurrent gastric cancer had a very limited role in the surveillance for tumor recurrence, but it is useful in the early detection and treatment of benign strictures of the anastomosis site, which does not improve survival rate but does improve the quality of life of patients [21].

In our study, in 70 EGC patients with TG with R0 resection, no tumor recurrence was found during mean follow-up periods of 42.1 ± 18 and 43.2 ± 19 months by endoscopy and contrast abdominal CT scan, respectively. However, in 197 AGC patients with TG with R0 resection, we found four cases of anastomosis site recurrence and two cases of duodenal stump and jejunal loop site recurrence. Among them, three cases of anastomosis site recurrence were found by both follow-up endoscopy and contrast abdominal CT scan, but only one case was detected by endoscopy and missed by contrast abdominal CT scan. In this case, we have reviewed the abdominal CT image with the radiologist again, but we heard that it is difficult to judge the tumor recurrence through this CT image. They said that it may be a problem related to CT resolution at that time. So, the reason for missing it on contrast abdominal CT scan may be that the tumor recurred as a slightly elevated mucosa and hyperemic erosion, difficult to observe on contrast abdominal CT scan.

In our study, we found that the clinical efficacy of periodic follow-up endoscopy for detection of recurred gastric cancer is very low, especially in patients with EGC. Although recurrent gastric cancers around the anastomosis site were detected by follow-up endoscopy, the overall survival rate of patients depends on their age, comorbidity, performance status, and availability of an effective treatment modality.

In Korea, endoscopy is performed every two years for adults over 40 years of age under the control of the insurance corporation. In most cases, patients who do not suspect tumor recurrence after 5 years of TG are receiving endoscopy every 2 years like other adults over 40 years of age. So we would like to suggest, in this study, if the recurrence of cancer is not strongly suspected, or if the uncomfortable symptoms do not happened, such as regurgitation, heartburn etc, after TG, it is not cost effective to perform annual follow-up endoscopic surveillance with abdominal CT, especially in patients diagnosed with EGC.

There are several limitations to our study. First, the study is retrospective and the number of patients enrolled is small. Of 747 patients, only 267 were included, the reason for this is as follows. Most other patients who were not included in this study received TG only from our hospital, and the necessary follow-up outpatient observation and other treatments (e.g. CTx or RTx. etc) were received at the hospital near the residence. So they were not included in the outpatient surveillance and observation of our hospital and so were not included in this study. This seems to be due to the special medical needs of Koreans (most Koreans want to receive a surgery at large and famous university hospital). So a large, prospective, multi-center study is necessary to confirm the findings. But it may be very difficult to conduct randomized controlled trial (RCT) in terms of deciding the study period, the size of the patients involved and solving the ethical issues that may arise. In addition, the absence of existing research literature is also one of the difficulties to establish and perform RCT. Therefore, we suggest the following realistically feasible research plan to further support our results. If we compare the patient group who underwent annually follow-up endoscopy and abdominal CT in the hospital after TG and the patients who underwent bi-annually endoscopy at the Korean health insurance corporation without any follow-up examinations at the hospital for any reason. It is meaningful way to actually check the clinical efficacy of annually follow-up endoscopy. Second, we do not know if the patients had symptoms or complaints when they underwent a follow-up endoscopy, because many endoscopists have simply described the chief complaint as a routine check-up after the gastric cancer surgery in the patient’s endoscopic records. In future, we need to record the patient’s complaints and symptoms when they receive follow-up endoscopy.

## Conclusions

Annual follow-up endoscopic surveillance for gastric cancer after TG might have a limited role in the detection of tumor recurrence, especially in patients diagnosed with EGC. Contrast abdominal CT scan may be sufficient as a follow-up method for recurrent gastric cancer after TG; endoscopy may only be necessary for patients who complain about specific symptoms due to stenosis of the anastomosis site.
